# Supplementary material for: Local read haplotagging enables accurate long-read small variant calling
Source: Nat Commun. 2024 Jul 13;15:5907. doi: 10.1038/s41467-024-50079-5 (PMC11246426; doi:10.1038/s41467-024-50079-5)
Supplement: Supplementary file 1 — Supplementary Information [file 41467_2024_50079_MOESM1_ESM.pdf]

Local read haplotagging enables accurate long-read small variant calling

Supplementary Notes

Commands used for analysis

pbmm2

We used pbmm2 to align PacBio reads to the reference. Following is the command used:

```
docker run -it -v /data:/data \
quay.io/biocontainers/pbmm2:1.10.0--h9ee0642_0 \
pbmm2 align --preset HIFI --sort \
/data/Reference.fasta \
/data/inputs.fofn \
/data/output.pbmm2.bam
```

minimap2

We used minimap2 to align ONT reads to the reference. Following is the command used:

```
minimap2 -k 17 -ax map-ont \
-t 95 REF.fasta INPUT.fastq.gz | samtools sort -@4 -m 4G > OUTPUT.bam
```

WhastHap

We used WhastHap to phase and haplotag PacBio and ONT data. Following is the command used:

```
whatshap phase --ignore-read-groups \
-o OUTPUT.phased.vcf -r REF.fasta \
INPUT.unphased.vcf INPUT.unhaplotagged.bam

whatshap haplotag \
--output OUTPUT.haplotagged.bam \
--reference /data/REF.fasta --ignore-read-groups \
OUTPUT.phased.vcf INPUT.unhaplotagged.bam
```

DeepVariant

We used DeepVariant variant caller to generate variant calls for PacBio and ONT data. Following is the command used:

```
docker run -v /data:/data \
google/deepvariant:1.5.0 \
/opt/deepvariant/bin/run_deepvariant \
--model_type=PACBIO \
--ref=/data/REF.fasta \
--reads=/data/INPUT.bam \
--output_vcf=/data/OUTPUT.vcf \
--output_gvcf=/data/OUTPUT.gvcf \
--num_shards=95 \
--logging_dir=/data/log_dir
```

We used `google/deepvariant:1.5.0` for analysis that includes approximate haplotagging and `google/deepvariant:1.2.0` for baseline comparison with no haplotagging and haplotagging with WhatsHap analysis. We used `-model_type=PACBIO` for variant calling on PacBio data and `-model_type=ONT_R104` for variant calling with ONT data.

Clair3

We used `Clair3` variant caller to generate variant calls for ONT data. Following is the command used:

```
sudo docker run -it -v /data:/data \
hkubal/clair3:latest \
/opt/bin/run_clair3.sh \
--bam_fn=/data/INPUT.bam \
--ref_fn=/data/REF.fasta \
--threads=95 \
--platform="ont" \
--model_path=/data/model_path/ \
--output=/data/output_dir/
```

We used `r1041_e82_400bps_sup_g615` model for variant calling R10.4 chemistry data with simplex and duplex types.

PEPPER

We used `PEPPER` variant caller to generate variant calls for ONT data. Following is the command used:

```
time docker run -it -v /data:/data \
kishwars/pepper_deepvariant:r0.8 \
run_pepper_margin_deepvariant call_variant \
-b /data/INPUT.bam \
-f /data/REF.fasta \
-o /data/output_dir/ \
-p output_prefix \
-t 95 \
--ont_r9_guppy5_sup
```

Hap.py

We used `hap.py` version `v0.3.12` to compare variant calls against GIAB truth set. Following is the command used:

```
docker run -it -v /data/:/data/ \
jmcdani20/hap.py:v0.3.12 /opt/hap.py/bin/hap.py \
/data/Benchmark.vcf.gz \
/data/INPUT.vcf.gz \
-f /data/Benchmark.bed \
-r /data/reference.fna \
-o /data/output/prefix \
--pass-only \
--engine=vcfeval \
--threads=95
```

BEST

We used best software `https://github.com/google/best` to assess the quality of the reads against reference genome.

```
./best \
INPUT.bam REF.fasta \
OUTPUT_DIR/OUTPUT_PREFIX \
--intervals-bed INTERVAL.bed -t 96
```

Supplementary Figures

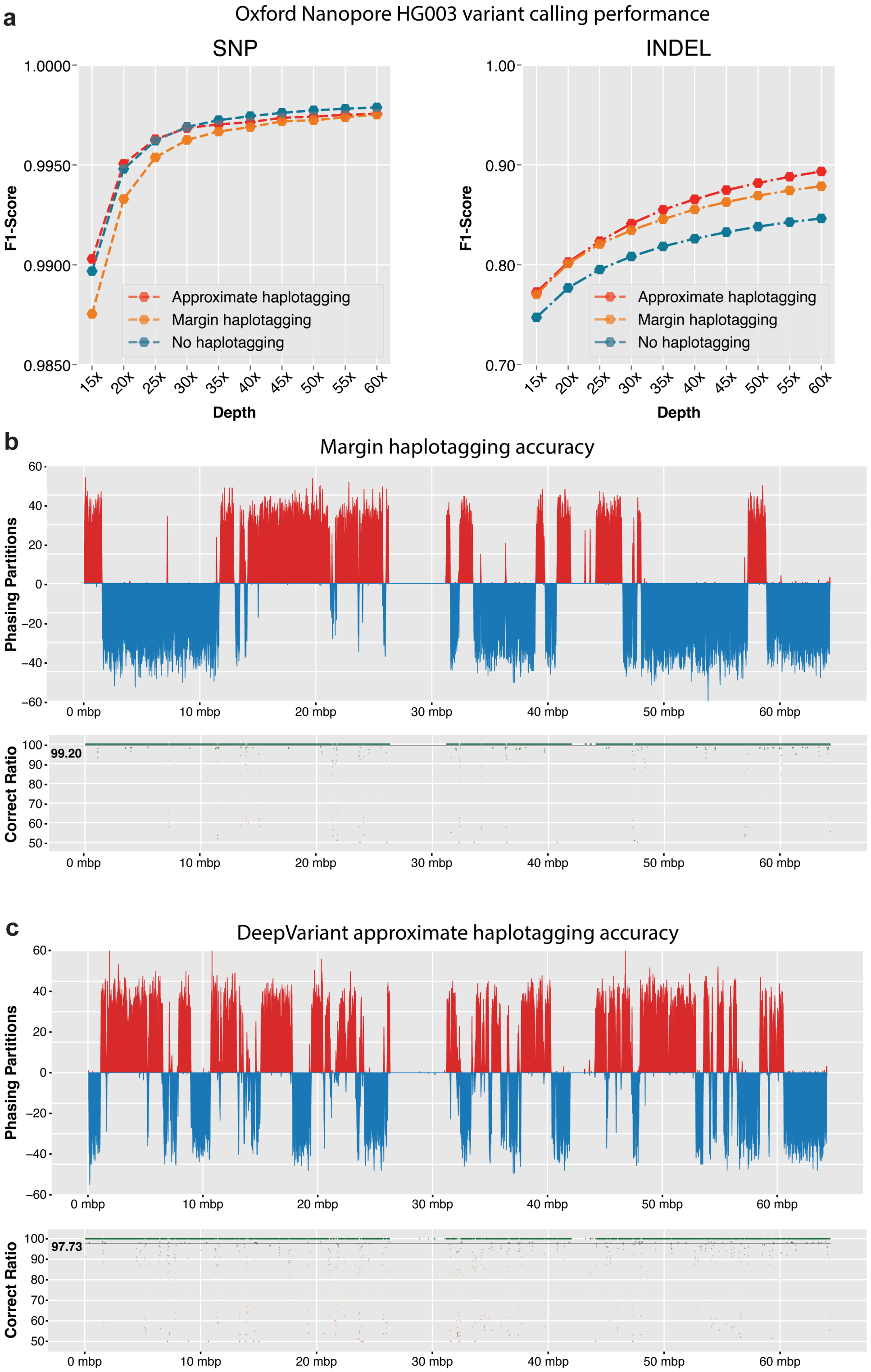

Supplementary Figure 1: Effectiveness of local haplotagging approximation for variant calling with Oxford Nanopore data. **a.** Variant calling accuracy improvement with approximate haplotaaging. **b.** Haplotagging accuracy of Margin. **c.** Haplotagging accuracy with the local approximate haplotagging method.

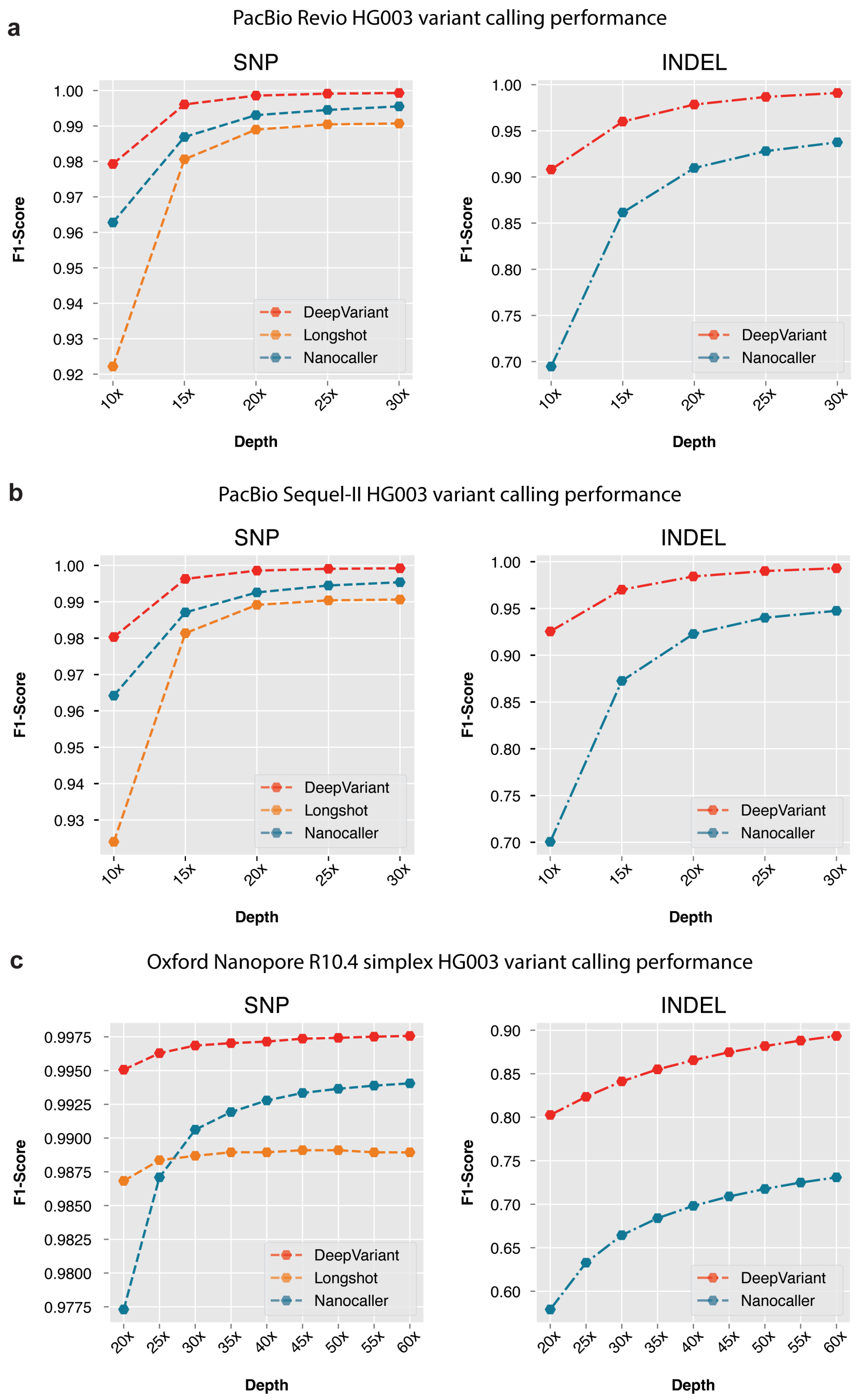

Supplementary Figure 2: Variant calling accuracy comparison between different variant callers DeepVariant, LongShot and NanoCaller. **a.** PacBio Revio variant calling performance comparison. **b.** PacBio Sequel-II variant calling performance comparison. **c.** ONT R10.4 simplex variant calling performance comparison.

Supplementary Tables

Coverage	Mode	Type	Total	TP	FN	FP	Recall	Precision	F1-Score
15x	No haplotag information	INDEL	504501	475924	28577	19110	0.943356	0.962773	0.952966
		SNP	3327495	3306618	20877	5629	0.993726	0.998302	0.996009
	WhatsHap haplotagging	INDEL	504501	487406	17095	12968	0.966115	0.975029	0.970551
		SNP	3327495	3306574	20921	4750	0.993713	0.998567	0.996134
	Approximate haplotagging	INDEL	504501	486509	17992	12463	0.964337	0.975933	0.970101
		SNP	3327495	3307835	19660	4831	0.994092	0.998543	0.996312
20x	No haplotag information	INDEL	504501	488680	15821	12121	0.96864	0.976708	0.972658
		SNP	3327495	3319839	7656	3549	0.997699	0.998933	0.998316
	WhatsHap haplotagging	INDEL	504501	496068	8433	7620	0.983284	0.985451	0.984366
		SNP	3327495	3319757	7738	2778	0.997675	0.999165	0.998419
	Approximate haplotagging	INDEL	504501	495573	8928	7237	0.982303	0.986157	0.984227
		SNP	3327495	3320920	6575	2814	0.998024	0.999154	0.998589
25x	No haplotag information	INDEL	504501	494504	9997	8298	0.980184	0.984133	0.982155
		SNP	3327495	3322610	4885	2911	0.998532	0.999125	0.998829
	WhatsHap haplotagging	INDEL	504501	499415	5086	4981	0.989919	0.99051	0.990214
		SNP	3327495	3322526	4969	2245	0.998507	0.999325	0.998916
	Approximate haplotagging	INDEL	504501	499064	5437	4789	0.989223	0.990867	0.990044
		SNP	3327495	3323614	3881	2231	0.998834	0.99933	0.999082
30x	No haplotag information	INDEL	504501	497618	6883	5985	0.986357	0.988581	0.987468
		SNP	3327495	3323336	4159	2521	0.99875	0.999243	0.998996
	WhatsHap haplotagging	INDEL	504501	501098	3403	3408	0.993255	0.993513	0.993384
		SNP	3327495	3323326	4169	1889	0.998747	0.999432	0.99909
	Approximate haplotagging	INDEL	504501	500790	3711	3481	0.992644	0.99337	0.993007
		SNP	3327495	3324370	3125	1989	0.999061	0.999403	0.999232
35x	No haplotag information	INDEL	504501	499364	5137	4469	0.989818	0.991481	0.990649
		SNP	3327495	3323559	3936	2257	0.998817	0.999322	0.999069
	WhatsHap haplotagging	INDEL	504501	501845	2656	2735	0.994735	0.994795	0.994765
		SNP	3327495	3323619	3876	1830	0.998835	0.99945	0.999143
	Approximate haplotagging	INDEL	504501	501629	2872	2771	0.994307	0.994725	0.994516
		SNP	3327495	3324633	2862	1852	0.99914	0.999444	0.999292

Supplementary Table 1: PacBio-HiFi variant calling performance of DeepVariant with no haplotagging, whatshap haplotagging and approximate haplotagging.

Coverage	Platform	Type	Total	TP	FN	FP	Recall	Precision	F1_Score
5x	Sequel II	INDEL	504501	325065	179436	48949	0.64433	0.87124	0.740798
		SNP	3327495	2568343	759152	119685	0.771855	0.955498	0.853914
	Revio	INDEL	504501	314909	189592	57900	0.624199	0.847147	0.718782
		SNP	3327495	2565892	761603	119109	0.771118	0.955662	0.853529
10x	Sequel II	INDEL	504501	454954	49547	24525	0.90179	0.950427	0.92547
		SNP	3327495	3216400	111095	18094	0.966613	0.99441	0.980315
	Revio	INDEL	504501	445946	58555	32760	0.883935	0.933607	0.908092
		SNP	3327495	3211531	115964	19959	0.96515	0.993828	0.979279
15x	Sequel II	INDEL	504501	486509	17992	12463	0.964337	0.975933	0.970101
		SNP	3327495	3307835	19660	4831	0.994092	0.998543	0.996312
	Revio	INDEL	504501	480852	23649	17008	0.953124	0.967055	0.960039
		SNP	3327495	3306551	20944	5121	0.993706	0.998455	0.996075
20x	Sequel II	INDEL	504501	495573	8928	7237	0.982303	0.986157	0.984227
		SNP	3327495	3320920	6575	2814	0.998024	0.999154	0.998589
	Revio	INDEL	504501	492207	12294	9771	0.975631	0.981271	0.978443
		SNP	3327495	3320738	6757	2801	0.997969	0.999158	0.998563
25x	Sequel II	INDEL	504501	499064	5437	4789	0.989223	0.990867	0.990044
		SNP	3327495	3323614	3881	2231	0.998834	0.99933	0.999082
	Revio	INDEL	504501	497096	7405	6223	0.985322	0.988116	0.986717
		SNP	3327495	3323686	3809	2059	0.998855	0.999381	0.999118
30x	Sequel II	INDEL	504501	500790	3711	3481	0.992644	0.99337	0.993007
		SNP	3327495	3324370	3125	1989	0.999061	0.999403	0.999232
	Revio	INDEL	504501	499552	4949	4354	0.99019	0.991699	0.990944
		SNP	3327495	3324580	2915	1727	0.999124	0.999481	0.999303

Supplementary Table 2: PacBio-HiFi variant calling performance comparison of DeepVariant on Sequel-II and Revio platforms at different coverages.

Cov.	Caller	Type	Total	TP	FN	FP	Recall	Precision	F1
10x	DeepVariant	INDEL	504501	316320	188181	69165	0.626996	0.824024	0.712133
		SNP	3327495	3152000	175495	56238	0.947259	0.982476	0.964546
	PEPPER	INDEL	504501	298224	206277	48609	0.591127	0.862071	0.70134
		SNP	3327495	3140268	187227	62700	0.943733	0.980428	0.961731
	Clair3	INDEL	504501	334539	169962	132794	0.663109	0.719643	0.69022
		SNP	3327495	3256496	70999	225280	0.978663	0.935315	0.956498
15x	DeepVariant	INDEL	504501	359668	144833	69022	0.712918	0.843013	0.772527
		SNP	3327495	3288775	38720	25744	0.988364	0.992235	0.990296
	PEPPER	INDEL	504501	350018	154483	55450	0.69379	0.865762	0.770294
		SNP	3327495	3283461	44034	38814	0.986767	0.98832	0.987543
	Clair3	INDEL	504501	365993	138508	86500	0.725455	0.812119	0.766345
		SNP	3327495	3305838	21657	58142	0.993492	0.982723	0.988078
20x	DeepVariant	INDEL	504501	380627	123874	65512	0.754462	0.857262	0.802584
		SNP	3327495	3312923	14572	18308	0.995621	0.994506	0.995063
	PEPPER	INDEL	504501	374497	130004	57136	0.742312	0.870199	0.801184
		SNP	3327495	3311580	15915	28778	0.995217	0.991387	0.993298
	Clair3	INDEL	504501	382081	122420	66293	0.757344	0.854966	0.8032
		SNP	3327495	3316247	11248	31521	0.99662	0.990588	0.993595
25x	DeepVariant	INDEL	504501	395117	109384	62184	0.783184	0.868111	0.823463
		SNP	3327495	3318240	9255	15473	0.997219	0.99536	0.996289
	PEPPER	INDEL	504501	389165	115336	55945	0.771386	0.876844	0.820741
		SNP	3327495	3318979	8516	22297	0.997441	0.993329	0.99538
	Clair3	INDEL	504501	393418	111083	56896	0.779816	0.876246	0.825223
		SNP	3327495	3319621	7874	24503	0.997634	0.992676	0.995149
30x	DeepVariant	INDEL	504501	407454	97047	58882	0.807638	0.877687	0.841207
		SNP	3327495	3320004	7491	13466	0.997749	0.995962	0.996854
	PEPPER	INDEL	504501	399453	105048	54650	0.791778	0.882125	0.834514
		SNP	3327495	3321557	5938	19046	0.998215	0.9943	0.996254
	Clair3	INDEL	504501	401819	102682	51547	0.796468	0.88874	0.840078
		SNP	3327495	3320762	6733	21388	0.997977	0.993603	0.995785
35x	DeepVariant	INDEL	504501	416788	87713	55671	0.826139	0.885965	0.855007
		SNP	3327495	3320458	7037	12739	0.997885	0.99618	0.997032
	PEPPER	INDEL	504501	407403	97098	52990	0.807537	0.88734	0.845559
		SNP	3327495	3322620	4875	17284	0.998535	0.994826	0.996677
	Clair3	INDEL	504501	408248	96253	47749	0.809211	0.897618	0.851125
		SNP	3327495	3321463	6032	18754	0.998187	0.994388	0.996284

Supplementary Table 3: Oxford Nanopore Technologies variant calling performance comparison between DeepVariant, PEPPER and Clair3 at different coverages between 10x to 35x.

Cov.	Caller	Type	Total	TP	FN	FP	Recall	Precision	F1
40x	DeepVariant	INDEL	504501	423764	80737	52909	0.839967	0.892638	0.865502
		SNP	3327495	3320622	6873	12133	0.997934	0.996361	0.997147
	PEPPER	INDEL	504501	414119	90382	50919	0.820849	0.892862	0.855342
		SNP	3327495	3323047	4448	16226	0.998663	0.995142	0.9969
	Clair3	INDEL	504501	413098	91403	44926	0.818825	0.90416	0.859379
		SNP	3327495	3321714	5781	16813	0.998263	0.994966	0.996612
45x	DeepVariant	INDEL	504501	429614	74887	50066	0.851562	0.899097	0.874684
		SNP	3327495	3320801	6694	10871	0.997988	0.996738	0.997363
	PEPPER	INDEL	504501	419218	85283	49312	0.830956	0.897071	0.862748
		SNP	3327495	3323348	4147	14599	0.998754	0.995628	0.997188
	Clair3	INDEL	504501	416625	87876	42430	0.825816	0.90973	0.865744
		SNP	3327495	3322293	5202	12983	0.998437	0.996109	0.997272
50x	DeepVariant	INDEL	504501	434092	70409	47794	0.860438	0.904207	0.88178
		SNP	3327495	3320812	6683	10474	0.997992	0.996857	0.997424
	PEPPER	INDEL	504501	423610	80891	47830	0.839661	0.90081	0.869161
		SNP	3327495	3323553	3942	14460	0.998815	0.995669	0.99724
	Clair3	INDEL	504501	419323	85178	40845	0.831164	0.913352	0.870322
		SNP	3327495	3322322	5173	12292	0.998445	0.996315	0.997379
55x	DeepVariant	INDEL	504501	438117	66384	45778	0.868417	0.908663	0.888084
		SNP	3327495	3320863	6632	9938	0.998007	0.997017	0.997512
	PEPPER	INDEL	504501	427106	77395	46546	0.846591	0.903942	0.874327
		SNP	3327495	3323552	3943	13473	0.998815	0.995964	0.997387
	Clair3	INDEL	504501	421823	82678	39301	0.836119	0.91683	0.874616
		SNP	3327495	3322252	5243	11429	0.998424	0.996573	0.997498
60x	DeepVariant	INDEL	504501	441442	63059	43947	0.875007	0.912632	0.893423
		SNP	3327495	3320861	6634	9573	0.998006	0.997127	0.997566
	PEPPER	INDEL	504501	430032	74469	45458	0.852391	0.906561	0.878642
		SNP	3327495	3323667	3828	12639	0.99885	0.996213	0.997529
	Clair3	INDEL	504501	423735	80766	37991	0.839909	0.91973	0.878009
		SNP	3327495	3322068	5427	9920	0.998369	0.997024	0.997696
65x	DeepVariant	INDEL	504501	444208	60293	42612	0.88049	0.915553	0.897679
		SNP	3327495	3320812	6683	9294	0.997992	0.99721	0.997601
	PEPPER	INDEL	504501	432767	71734	43959	0.857812	0.909908	0.883093
		SNP	3327495	3323726	3769	11211	0.998867	0.996639	0.997752
	Clair3	INDEL	504501	424915	79586	37232	0.842248	0.921413	0.880054
		SNP	3327495	3321510	5985	9341	0.998201	0.997197	0.997699

Supplementary Table 4: Oxford Nanopore Technologies variant calling performance comparison between DeepVariant, PEPPER and Clair3 at different coverages between 40x to 65x.

Cov.	Platform	Type	Total	TP	FN	FP	Recall	Precision	F1
10x	R10.4.1 DeepVariant	INDEL	504501	316320	188181	69165	0.626996	0.824024	0.712133
		SNP	3327495	3152000	175495	56238	0.947259	0.982476	0.964546
	R9.4.1 PEPPER	INDEL	504501	209342	295159	40606	0.414949	0.839909	0.555472
		SNP	3327495	2984659	342836	57620	0.896969	0.981062	0.937133
15x	R10.4.1 DeepVariant	INDEL	504501	359668	144833	69022	0.712918	0.843013	0.772527
		SNP	3327495	3288775	38720	25744	0.988364	0.992235	0.990296
	R9.4.1 PEPPER	INDEL	504501	276696	227805	50025	0.548455	0.849811	0.666659
		SNP	3327495	3230875	96620	18904	0.970963	0.994184	0.982436
20x	R10.4.1 DeepVariant	INDEL	504501	380627	123874	65512	0.754462	0.857262	0.802584
		SNP	3327495	3312923	14572	18308	0.995621	0.994506	0.995063
	R9.4.1 PEPPER	INDEL	504501	311062	193439	53260	0.616574	0.856908	0.717141
		SNP	3327495	3286111	41384	11946	0.987563	0.996379	0.991951
25x	R10.4.1 DeepVariant	INDEL	504501	395117	109384	62184	0.783184	0.868111	0.823463
		SNP	3327495	3318240	9255	15473	0.997219	0.99536	0.996289
	R9.4.1 PEPPER	INDEL	504501	332679	171822	52935	0.659422	0.865802	0.748649
		SNP	3327495	3303138	24357	9843	0.99268	0.99703	0.99485
30x	R10.4.1 DeepVariant	INDEL	504501	407454	97047	58882	0.807638	0.877687	0.841207
		SNP	3327495	3320004	7491	13466	0.997749	0.995962	0.996854
	R9.4.1 PEPPER	INDEL	504501	348343	156158	51857	0.69047	0.873415	0.771242
		SNP	3327495	3310086	17409	8939	0.994768	0.997307	0.996036
35x	R10.4.1 DeepVariant	INDEL	504501	416788	87713	55671	0.826139	0.885965	0.855007
		SNP	3327495	3320458	7037	12739	0.997885	0.99618	0.997032
	R9.4.1 PEPPER	INDEL	504501	360393	144108	50461	0.714355	0.880074	0.788603
		SNP	3327495	3313305	14190	8230	0.995736	0.997523	0.996628

Supplementary Table 5: Oxford Nanopore Technologies variant calling performance comparison between R9.4.1 and R10.4 chemistry data at coverages between 10x and 35x.

Coverage	Platform	Type	Total	TP	FN	FP	Recall	Precision	F1_Score
40x	R10.4.1	INDEL	504501	423764	80737	52909	0.839967	0.892638	0.865502
	DeepVariant	SNP	3327495	3320622	6873	12133	0.997934	0.996361	0.997147
	R9.4.1	INDEL	504501	370622	133879	49181	0.734631	0.885651	0.803103
	PEPPER	SNP	3327495	3315180	12315	7993	0.996299	0.997595	0.996947
45x	R10.4.1	INDEL	504501	429614	74887	50066	0.851562	0.899097	0.874684
	DeepVariant	SNP	3327495	3320801	6694	10871	0.997988	0.996738	0.997363
	R9.4.1	INDEL	504501	378872	125629	47879	0.750984	0.890526	0.814824
	PEPPER	SNP	3327495	3316265	11230	7717	0.996625	0.997679	0.997152
50x	R10.4.1	INDEL	504501	434092	70409	47794	0.860438	0.904207	0.88178
	DeepVariant	SNP	3327495	3320812	6683	10474	0.997992	0.996857	0.997424
	R9.4.1	INDEL	504501	385925	118576	46668	0.764964	0.894782	0.824796
	PEPPER	SNP	3327495	3316884	10611	7169	0.996811	0.997844	0.997327
55x	R10.4.1	INDEL	504501	438117	66384	45778	0.868417	0.908663	0.888084
	DeepVariant	SNP	3327495	3320863	6632	9938	0.998007	0.997017	0.997512
	R9.4.1	INDEL	504501	392084	112417	45404	0.777172	0.898791	0.833569
	PEPPER	SNP	3327495	3317426	10069	7255	0.996974	0.997818	0.997396
60x	R10.4.1	INDEL	504501	441442	63059	43947	0.875007	0.912632	0.893423
	DeepVariant	SNP	3327495	3320861	6634	9573	0.998006	0.997127	0.997566
	R9.4.1	INDEL	504501	397171	107330	44010	0.787255	0.902738	0.841051
	PEPPER	SNP	3327495	3317700	9795	7126	0.997056	0.997857	0.997457
65x	R10.4.1	INDEL	504501	444208	60293	42612	0.88049	0.915553	0.897679
	DeepVariant	SNP	3327495	3320812	6683	9294	0.997992	0.99721	0.997601
	R9.4.1	INDEL	504501	401236	103265	42583	0.795313	0.906457	0.847255
	PEPPER	SNP	3327495	3317934	9561	7111	0.997127	0.997862	0.997494

Supplementary Table 6: Oxford Nanopore Technologies variant calling performance comparison between R9.4.1 and R10.4 chemistry data at coverages between 40x and 65x.

Cov	Type	Type	Total	TP	FN	FP	Recall	Precision	F1
10x	R10.4.1 Duplex	INDEL	11256	7610	3646	1649	0.676084	0.825207	0.743239
		SNP	71333	67511	3822	905	0.94642	0.986779	0.966178
	R10.4.1 Simplex	INDEL	11256	6751	4505	1614	0.599769	0.810073	0.689236
		SNP	71333	66281	5052	1336	0.929177	0.980252	0.954031
15x	R10.4.1 Duplex	INDEL	11256	8624	2632	1529	0.766169	0.853051	0.807279
		SNP	71333	70666	667	200	0.990649	0.997179	0.993904
	R10.4.1 Simplex	INDEL	11256	7842	3414	1572	0.696695	0.836454	0.760205
		SNP	71333	70054	1279	533	0.98207	0.992453	0.987234
20x	R10.4.1 Duplex	INDEL	11256	9092	2164	1383	0.807747	0.871397	0.838365
		SNP	71333	71184	149	113	0.997911	0.998416	0.998164
	R10.4.1 Simplex	INDEL	11256	8353	2903	1498	0.742093	0.851566	0.793069
		SNP	71333	70967	366	223	0.994869	0.996869	0.995868
25x	R10.4.1 Duplex	INDEL	11256	9359	1897	1312	0.831468	0.880488	0.855276
		SNP	71333	71250	83	93	0.998836	0.998697	0.998767
	R10.4.1 Simplex	INDEL	11256	8690	2566	1425	0.772033	0.862849	0.814918
		SNP	71333	71169	164	162	0.997701	0.99773	0.997716
30x	R10.4.1 Duplex	INDEL	11256	9528	1728	1282	0.846482	0.884868	0.865249
		SNP	71333	71269	64	79	0.999103	0.998893	0.998998
	R10.4.1 Simplex	INDEL	11256	8974	2282	1369	0.797264	0.871335	0.832655
		SNP	71333	71211	122	141	0.99829	0.998025	0.998157
35x	R10.4.1 Duplex	INDEL	11256	9700	1556	1162	0.861763	0.896453	0.878766
		SNP	71333	71276	57	78	0.999201	0.998907	0.999054
	R10.4.1 Simplex	INDEL	11256	9145	2111	1338	0.812456	0.876088	0.843073
		SNP	71333	71236	97	123	0.99864	0.998277	0.998459
40x	R10.4.1 Duplex	INDEL	11256	9830	1426	1068	0.873312	0.905109	0.888926
		SNP	71333	71279	54	73	0.999243	0.998978	0.99911
	R10.4.1 Simplex	INDEL	11256	9318	1938	1204	0.827825	0.888961	0.857304
		SNP	71333	71233	100	123	0.998598	0.998277	0.998438
45x	R10.4.1 Duplex	INDEL	11256	9920	1336	1011	0.881308	0.910523	0.895677
		SNP	71333	71283	50	69	0.999299	0.999033	0.999166
	R10.4.1 Simplex	INDEL	11256	9443	1813	1171	0.83893	0.892834	0.865043
		SNP	71333	71239	94	127	0.998682	0.998222	0.998452
50x	R10.4.1 Duplex	INDEL	11256	10024	1232	951	0.890547	0.916248	0.903215
		SNP	71333	71286	47	69	0.999341	0.999034	0.999187
	R10.4.1 Simplex	INDEL	11256	9530	1726	1108	0.84666	0.898914	0.872005
		SNP	71333	71249	84	120	0.998822	0.99832	0.998571

Supplementary Table 7: Oxford Nanopore Technologies variant calling performance comparison between Simplex and Duplex data types.
