# Supplementary material for: Local read haplotagging enables accurate long-read small variant calling
Source: Nat Commun. 2024 Jul 13;15:5907. doi: 10.1038/s41467-024-50079-5 (PMC11246426; doi:10.1038/s41467-024-50079-5)
Supplement: Supplementary file 3 — Reporting Summary [file 41467_2024_50079_MOESM3_ESM.pdf]

nature portfolio

Corresponding author(s): K.S., A.W.C.

Last updated by author(s): May 15, 2024

Reporting Summary

Nature Portfolio wishes to improve the reproducibility of the work that we publish. This form provides structure for consistency and transparency in reporting. For further information on Nature Portfolio policies, see our Editorial Policies and the Editorial Policy Checklist.

Statistics

For all statistical analyses, confirm that the following items are present in the figure legend, table legend, main text, or Methods section.

n/a	Confirmed	
☐	☒	The exact sample size (n) for each experimental group/condition, given as a discrete number and unit of measurement
☐	☒	A statement on whether measurements were taken from distinct samples or whether the same sample was measured repeatedly
☐	☒	The statistical test(s) used AND whether they are one- or two-sided<br>*Only common tests should be described solely by name; describe more complex techniques in the Methods section.*
☒	☐	A description of all covariates tested
☒	☐	A description of any assumptions or corrections, such as tests of normality and adjustment for multiple comparisons
☐	☒	A full description of the statistical parameters including central tendency (e.g. means) or other basic estimates (e.g. regression coefficient) AND variation (e.g. standard deviation) or associated estimates of uncertainty (e.g. confidence intervals)
☒	☐	For null hypothesis testing, the test statistic (e.g. F, t, r) with confidence intervals, effect sizes, degrees of freedom and P value noted<br>*Give P values as exact values whenever suitable.*
☒	☐	For Bayesian analysis, information on the choice of priors and Markov chain Monte Carlo settings
☒	☐	For hierarchical and complex designs, identification of the appropriate level for tests and full reporting of outcomes
☒	☐	Estimates of effect sizes (e.g. Cohen's d, Pearson's r), indicating how they were calculated

Our web collection on statistics for biologists contains articles on many of the points above.

Software and code

Policy information about availability of computer code

Data collection	All data used in this study are publicly available:<br>We have made all data including input BAMs, output VCF and analysis files publicly available: https://console.cloud.google.com/storage/browser/brain-genomics-public/publications/kolesnikov2023_dv_haplotagging/evaluation/.<br><br>Moreover, all data collected and used for this study are publicly available though the HPRC consortium:<br>https://s3-us-west-2.amazonaws.com/human-pangenomics/index.html
Data analysis	Details of data analysis methods is described in the data analysis section of the manuscript.<br>Read alignment and subsampling: We used pbmm2 version 1.10 and minimap2 version 2.24-r1122 to align reads to the reference genome. We used samtools version 1.15 for sampling alignment files at different coverages.<br>Variant calling and haplotagging: We used PEPPER-Margin-DeepVariant version r0.8, Clair3 version v1.0.0 for variant calling, for DeepVariant-WhatsHap-DeepVariant pipeline we used v1.2.0 version of DeepVariant. For haplotagging with WhatsHap, we used WhatsHap version v1.7.<br>Benchmarking variant calls: For benchmarking variant calls, we used hap.py version v0.3.12.<br>Haplotagging accuracy and natural switch determination: We used https://github.com/tpesout/genomics_scripts/haplotagging/stats.py to calculate the haplotagging accuracy.<br>Read accuracy estimation: We used Best version v0.1.0 for read accuracy analysis. For the analysis, we used GRCh38 as the reference to derive the empirical QV for each read.

For manuscripts utilizing custom algorithms or software that are central to the research but not yet described in published literature, software must be made available to editors and reviewers. We strongly encourage code deposition in a community repository (e.g. GitHub). See the Nature Portfolio guidelines for submitting code & software for further information.

Data

Policy information about availability of data

All manuscripts must include a data availability statement. This statement should provide the following information, where applicable:

- Accession codes, unique identifiers, or web links for publicly available datasets
- A description of any restrictions on data availability
- For clinical datasets or third party data, please ensure that the statement adheres to our policy

All data used in this study are publicly available:
We have made all data including input BAMs, output VCF and analysis files publicly available: https://console.cloud.google.com/storage/browser/brain-genomics-public/publications/kolesnikov2023_dv_haplotagging/evaluation/.

Moreover, all data collected and used for this study are publicly available though the HPRC consortium: https://s3-us-west-2.amazonaws.com/human-pangenomics/index.html. The required data policy and details of the data can be found in https://humanpangenome.org/data/ and https://www.ncbi.nlm.nih.gov/bioproject/730823.

Research involving human participants, their data, or biological material

Policy information about studies with human participants or human data. See also policy information about sex, gender (identity/presentation), and sexual orientation and race, ethnicity and racism.

Reporting on sex and gender	N/A
Reporting on race, ethnicity, or other socially relevant groupings	N/A
Population characteristics	N/A
Recruitment	N/A
Ethics oversight	Data available through human pangenome reference consortium

Note that full information on the approval of the study protocol must also be provided in the manuscript.

Field-specific reporting

Please select the one below that is the best fit for your research. If you are not sure, read the appropriate sections before making your selection.

☒ Life sciences ☐ Behavioural & social sciences ☐ Ecological, evolutionary & environmental sciences

For a reference copy of the document with all sections, see nature.com/documents/nr-reporting-summary-flat.pdf

Life sciences study design

All studies must disclose on these points even when the disclosure is negative.

Sample size	We used HG001-HG007 human cell line sequencing from HPRC consortium
Data exclusions	For each study HG003 was held out from training and only used for evaluation. We also hold out chromosome 20 from training.
Replication	N/A
Randomization	N/A
Blinding	N/A

Reporting for specific materials, systems and methods

We require information from authors about some types of materials, experimental systems and methods used in many studies. Here, indicate whether each material, system or method listed is relevant to your study. If you are not sure if a list item applies to your research, read the appropriate section before selecting a response.

Materials & experimental systems

n/a	Involved in the study
☒	☐ Antibodies
☒	☐ Eukaryotic cell lines
☒	☐ Palaeontology and archaeology
☒	☐ Animals and other organisms
☒	☐ Clinical data
☒	☐ Dual use research of concern
☒	☐ Plants

Methods

n/a	Involved in the study
☒	☐ ChIP-seq
☒	☐ Flow cytometry
☒	☐ MRI-based neuroimaging
